# 897. Implementation of Burn Nursing Insertion of Ultrasound-guided Peripheral IVs

**DOI:** 10.1093/jbcr/irag033.393

**Published:** 2026-04-06

**Authors:** Amanda Venable

**Affiliations:** Timothy J. Harnar Burn Center UMC Health System, Lubbock, TX

## Abstract

**Introduction:**

Vascular access in burn patients is a challenging aspect of care that affects the healthcare team and patients daily. Limited options for vascular access due to surgical procedure sites and the avoidance of burned skin contribute to the difficulty of vascular access in burn patients. Multiple IV attempts are sometimes required in burn patients increasing the nursing time spent on IV access. Decreased confidence in the clinician performing the vascular access procedure can cause patients and their family members to doubt the competence of the healthcare team.

Vascular access teams (VAT) often use ultrasound equipment to insert IVs. With proper training, using ultrasound equipment to guide IV insertion may reduce the number of IV attempts. Improper training for ultrasound-guided peripheral IV (USGIV) insertions may result in serious complications, including arterial placement, infiltrations, and extravasations.

**Methods:**

A vascular access training program was developed to provide education for burn nurses. The program was equivalent to the training used for the hospital VAT. Nurses were trained to insert peripheral IV catheters and long dwell peripheral catheters using USGIV. The program's success was monitored by tracking the number of attempts and VAT consults. Complications were monitored according to hospital policy. Nine nurses were trained over two years. Charts were reviewed at the program start in 2023 and two years into the program. The data collected included dwell time, attempts, and reason for catheter discontinuation. Three groups were compared in both time cohorts: VAT insertions, Burn USGIV-trained insertions, and nurse insertions with no USGIV training.

**Results:**

248 instances of peripheral IV catheter documentation were reviewed from 2023, and another 248 from 2025. The number of insertion attempts averaged 1.06 in both 2023 and 2025 for the VAT. Insertion attempts for Burn USGIV nurses was 1.04 in 2023 and 1.074 in 2025. The average attempts for nurses with no special training were 1.11 in 2023 and 1.16 in 2025. Documented long dwell peripheral IVs were all 1 attempt for VAT and Burn USGIV in both time cohorts. There was a 47% reduction in lines inserted by VAT once the training program matured and a 76.9% increase in insertions by a USGIV trained burn nurse. See table for more results.

**Conclusions:**

USGIV insertion performed by bedside staff nurses can be successfully implemented as a program with careful consideration of quality monitoring. Burn nurses can insert IVs on their own, even in complex cases, preventing potential delays in care while waiting for a VAT insertion.

**Applicability of Research to Practice:**

A USGIV program in a burn unit is a useful tool for increasing the availability of advanced peripheral vascular access practices for burn patients.

**Funding for the study:**

N/A.

